# Adjunctive Strategies in the Management of Resistant, ‘Undilatable’ Coronary Lesions After Successfully Crossing a CTO with a Guidewire

**DOI:** 10.2174/1573403X10666140331124954

**Published:** 2014-05

**Authors:** Sara L. Fairley, James C. Spratt, Omar Rana, Suneel Talwar, Colm Hanratty, Simon Walsh

**Affiliations:** 1 Cardiology Department, Belfast Health and Social Care Trust, BT9 7AB, UK;; 2 Cardiology Department, Forth Valley Royal Hospital, Stirling Road, Larbert, UK;; 3 Dorset Heart Centre, Royal Bournemouth Hospital, Bournemouth, BH7 7DW, UK

**Keywords:** Chronic total occlusions, dissection re-entry techniques, excimer coronary laser atherectomy, high speed rotational atherectomy, novel revascularisation techniques.

## Abstract

Successful revascularisation of chronic total occlusions (CTOs) remains one of the greatest challenges in the era of contemporary percutaneous coronary intervention (PCI). Such lesions are encountered with increasing frequency in current clinical practice. A predictable increase in the future burden of CTO management can be anticipated given the ageing population, increased rates of renal failure, graft failure and diabetes mellitus. Given recent advances and developments in CTO PCI management, successful recanalisation can be anticipated in the majority of procedures undertaken at high-volume centres when performed by expert operators.

Despite advances in device technology, the management of resistant, calcific lesions remains one of the greatest challenges in successful CTO intervention. Established techniques to modify calcific lesions include the use of high-pressure non-compliant balloon dilation, cutting-balloons, anchor balloons and high speed rotational atherectomy (HSRA). Novel approaches have proven to be safe and technically feasible where standard approaches have failed. A step-wise progression of strategies is demonstrated, from well-recognised techniques to techniques that should only be considered when standard manoeuvres have proven unsuccessful. These methods will be described in the setting of clinical examples and include use of very high-pressure non-compliant balloon dilation, intentional balloon rupture with vessel dissection or balloon assisted micro-dissection (BAM), excimer coronary laser atherectomy (ECLA) and use of HSRA in various ‘offlabel’ settings.

## INTRODUCTION

In the contemporary era of interventional cardiology, chronic total occlusions (CTOs) are encountered with increasing frequency. Data from the Canadian Multicentre Chronic Total Occlusions Registry cited a CTO prevalence rate of 18.4% in all patients undergoing non-urgent coronary angiography [1]. Given the ageing population, increasing rates of renal failure and diabetes mellitus, one can anticipate a further increase in the burden of chronic occlusion management in the future. In addition, re-interventions are frequently encountered in the post-coronary artery bypass graft (CABG) group of patients, where the incidence of CTOs is > 50% [2]. Documented benefits of successful CTO intervention include improved left ventricular function, reduced angina frequency and enhanced exercise capacity [3].The importance of complete revascularisation (CR) in multi-vessel coronary disease has been highlighted in several studies. In the New York State PCI database, CR was demonstrated to be a positive prognostic factor [4]. Conversely, incomplete revascularisation (IR) after PCI has been associated with adverse clinical outcomes [5].

The greatest obstacles to successful recanalisation are related to vessel calcification and collateral channel tortuosity. However, despite the challenges associated with CTO PCI, recanalisation rates of > 85% can now be expected at experienced high-volume centres [6]. In the hands of appropriately experienced operators, there are few angiographic exclusions to contemporary CTO PCI. This was highlighted in the FAST-CTO trial, where novel hybrid revascularisation approaches were studied in 147 patients with a previously failed CTO PCI attempt. With an emphasis on the use of dissection / re-entry techniques, successful recanalisation rates were 87% in a sub-group of 75 cases. The 30-day major adverse clinical event (MACE) rate was acceptable at 4.8% [7]. 

To facilitate successful CTO recanalisation, a strategic approach combining understanding of lesion characteristics, coronary anatomy and adjunctive techniques should be used. To enhance procedural outcomes and CTO recanalisation rates, the operator should be familiar in the use and handling of a range of techniques and devices. The successful revascularisation of resistant coronary lesions is dependent on methods that enhance lesion crossing and methods that enhance lesion modification (Tables **1** & **2**). Details of these approaches will be discussed further in the case examples. 

## WIRE TECHNIQUES AND STRATEGIES

In addition to strategies to facilitate lesion crossing and lesion modification, guidewire selection greatly influences the ability of an operator to successfully cross a lesion and wire the distal vessel true lumen. A variety of guidewires and supplementary manoeuvres specific to lesion subsets are available. Detailed discussions of advanced guidewire technology are beyond the scope of this text, but have led to increased rates of successful lesion crossing. It is generally recognised that simple, focal lesions can be effectively and safely wired by skilled operators. 

In the J-CTO trial, utilisation of advanced guidewires facilitated successful lesion crossing in 88.6% of CTO cases [8]. All CTO operators should be familiar with the handling of a range of guidewires (including stiff, tapered CTO-specific wires), as appropriate selection may influence revascularisation success rates. It should be remembered that CTO-specific guidewires are more prone to enter sub-intimal channels, and may increase the risk of perforation. In one study, unintentional sub-intimal wire tracking occurred in 45% of CTO lesions as documented by intravascular ultrasound (IVUS) assessment [9]. 

More recently, advances in antegrade or retrograde wiring with dissection and re-entry techniques have afforded the opportunity of high procedural success rates with limited complications. Such techniques are facilitated by novel equipment including the CrossBoss™ and Stingray™ (Boston Scientific, Natick, MA) devices. Further details of these devices plus a discussion of retrograde wiring techniques will be considered in companion articles in this issue of the journal. In cases where crossing the proximal cap is found to be extremely challenging or impossible with standard techniques, deliberate bypassing of the cap by entering the sub-intimal space and performing antegrade or retrograde dissection, may facilitate a successful outcome. This is known as controlled antegrade and retrograde sub-intimal tracking (CART) [10]. These methods are described in detail in companion articles in this publication. Nevertheless, this option can be considered under appropriate circumstances. 

## METHODS TO FACILITATE LESION CROSSING

### a) Anchor Balloon Support Techniques

Anchor balloon support techniques remain a robust method to facilitate crossing of at least moderately resistant coronary lesions. Typically, proximal anchor balloon inflation in a side-branch enhances guide catheter stability and increases support prior to crossing the occlusion with a guidewire, micro-catheter or balloon [11]. Care should be taken with proximal anchor balloons, and over-sizing of the balloon diameter should be avoided to prevent side-branch disruption or perforation. Arrhythmias or significant ischaemia can be precipitated, although in our experience these events are very rare. In addition to placing an anchor balloon in a side-branch, it is also possible to trap a micro-catheter (usually a Corsair; Asahi Intecc, Japan) within the main vessel to facilitate extreme support for guidewire advancement through the occluded segment. When 8F guide catheters are used it is possible to use an anchor balloon in the side-branch as well as the main vessel with a Corsair, thus providing extreme support. If a lesion is successfully crossed, balloon inflation distal to, or even within the target lesion can also facilitate delivery of secondary equipment [12]. This distal balloon anchoring technique has also been described and used as an adjunct for the delivery of secondary equipment. 

### b) Extension Catheters

The introduction of extension catheters increased the success rate of lesion crossing by improving guide catheter support. The initial, low-profile Terumo Heartrail II (Terumo, Japan) catheter had relatively limited clinical utility due to its smaller lumen capacity, greater catheter length and subsequent requirement for long wire exchanges. More recently, the GuideLiner™ catheter (Vascular Solutions, Maple Grove, MN) was developed as a monorail or rapid-exchange mother and daughter system. The GuideLiner™ consists of a short guide-catheter extension connected to an introducer rod. The major advantage of the device is that is can be safely engaged very deeply in the distal vessel over an anchor balloon to allow stent delivery. In one of the first studies assessing its clinical utility, the device success rate was 93% in a series of 70 PCI cases. Of note, the greatest benefit was found in those cases with tortuous anatomy and complex, calcified lesions [13]. For this reason, it has become an established treatment modality in contemporary PCI.

### c) Micro-catheters

Micro-catheters were specifically developed to enhance lesion crossing and can substantially enhance the support offered to the guidewire when they are engaged within the CTO. A range of micro-catheters are available including the Quickcross (Spectranetics Corp, Colorado Springs, CO), Finecross (Terumo, Japan), NHancer (Interventional Medical Device Solutions, Netherlands), Corsair and Tornus (Asahi Intecc, Japan) devices. The Tornus and Corsair systems are manufactured from metallic alloys allowing the operator to cross difficult lesions and/or dilate collateral channels whilst being kink and compression resistant. Both of these micro-catheters can be used in conjunction with anchor balloon support to further enhance lesion crossing.

### Tornus (Asahi Intecc, Japan)

The Tornus was introduced in 2004 [14] to facilitate lesion crossing in those cases where the guidewire had only partially penetrated the lesion cap [12]. The device consists of a stainless steel, wire braided catheter (either 2.1F or 2.6F). It has a tapered steel tip to increase penetration and a silicone coating to increase lubricity. After the Tornus has crossed a lesion, this facilitates successful passage of low-profile balloons whilst minimizing the risk of vessel dissection or disruption. It can also be used to increase support to facilitate guidewire advancement [15]. The clinical utility of the device has been demonstrated in several studies, with successful revascularisation following its use in 91% cases of a cohort of 44 PCI patients [16], and 71.4% of a cohort of 56 severely calcified CTO lesions [17]. 

### Corsair (Asahi Intecc, Japan)

The development of the Corsair micro-catheter has complimented the use of the Tornus, as it has additional benefits as a collateral channel dilator. The Corsair is a braided micro-catheter consisting of 10 interwoven wires which form a polymer-covered metallic tube. The device has a kink-resistant, tapered tip to ease access to complex channels. The device improves wire support and wire manipulation. The Corsair also has the added advantage of allowing contrast injection through the catheter to delineate collateral channels. The Corsair will also frequently cross resistant lesions with or without balloon anchor support.

## METHODS TO FACILITATE LESION MODIFICATION

Calcified CTO segments present specific obstacles to successful revascularisation. The nature of the fibro-calcific plaque reduces vessel distensibility, making it resistant to aggressive balloon dilation. This increases the risk of stent under-expansion [18], late in-stent restenosis and adverse clinical events. Once the lesion has been successfully crossed, further challenges related to lesion modification can arise. A spectrum of techniques with escalating complexity can be considered when faced with challenging lesion modification (Table **2**). These strategies will be further discussed in the setting of case-based examples. It must be highlighted that these measures should be considered ‘last resort’ measures where standard techniques have proved unsuccessful. The descriptions of device use in the cases of balloon assisted micro-dissection (BAM), excimer laser coronary atherectomy (ELCA) and high speed rotational atherectomy (HSRA) in these CTO cases are all ‘off-license’ settings and are contraindicated in “Instructions for Use” documents. Therefore, such techniques should be performed by experienced CTO operators familiar with these advanced techniques, specific device use, potential complications and the management of complications should they arise.

### a) Very High-pressure Non-compliant Balloon Dilation

Very-high pressure balloon dilation is the most frequently employed strategy to manage resistant lesions. Non-compliant (NC) balloons facilitate lesion modification by delivering high intra-luminal pressures. In addition to standard non-compliant balloons, ultra non-compliant balloons such as the Schwager OPN balloon (SIS Medical, Switzerland), have been developed to deliver high post-dilation pressures of >40 atm. Operators must remember that there is an increased risk of vessel perforation when utilising very high pressure post-dilation strategies.

**Fig. (1):**
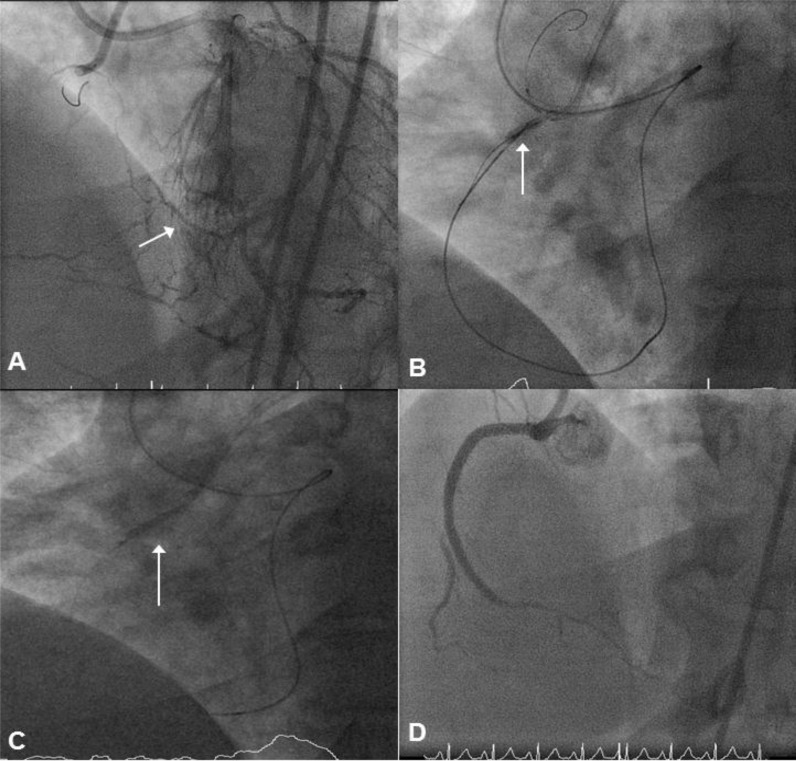
Panel A: There is a proximal CTO of the RCA with multiple septal collaterals from the LAD (arrow). Panel B: An antegrade Progress wire has successfully crossed the proximal CTO cap. A retrograde Pilot 200 wire has been advanced through the septal collaterals of the LAD to the chronic occlusion with assistance of a Corsair micro-catheter. Sequential antegrade balloon dilations have been performed to connect the sub-intimal channel (arrow). Panel C: A heavily calcified proximal RCA lesion persists, despite pre-dilation. This resistant area is further modified with a high-pressure non-compliant balloon at 30atm. Panel D: The vessel is then reconstructed using overlapping Promus Element DES (2.5/18, 3/12, 3/38).

Case example: A 41-year old ex-smoker with previous left anterior descending (LAD) artery stenting presented with a CTO of the proximal right coronary artery (RCA). There was no proximal or distal cap ambiguity, the occlusion was long (50mm), and the septal collaterals to the distal RCA were usable. The chosen interventional strategy was a primary retrograde approach. A Sion wire (Asahi Intecc, Japan) was advanced retrogradely to the distal RCA via the 2^nd^ septal branch. The Sion wire was exchanged for a Pilot 200 wire (Abbott Vascular, Santa Clara, CA) facilitating advancement of a Corsair micro-catheter to the mid-RCA. The antegrade channel was accessed with a Progress 200T wire (Abbott Vascular, Santa Clara, CA) with anchor balloon support. Sequential antegrade balloon dilations were performed to connect the sub-intimal spaces (reverse CART). A heavily calcified proximal RCA lesion persisted, and was modified with repeated high pressure inflations at 30 atm using a 3.0 Quantum NC balloon (Boston Scientific, Natick, MA). The retrograde Pilot 200 wire was advanced into the RCA guide, exchanged for an RG3 (Asahi Intecc, Japan), and externalised. The vessel was reconstructed with Promus Element stents (Boston Scientific, Natick, MA) and post-dilated with non-compliant balloons (3.0 in main lumen; 4.0 at the ostium). In this case, very high pressure inflation with a non-compliant balloon successfully modified the resistant lesion (Fig. **1**).

### b) Intentional Balloon Rupture / Balloon Assisted Micro-dissection (BAM)

The primary intention with BAM is to cause intentional and controlled vessel dissection, thus facilitating delivery of secondary equipment beyond the calcific lesion. This is an ‘off license’ technique and should be regarded as a ‘last resort’ measure where other strategies have failed. In the majority of cases, a very small diameter balloon is used (1.2 or 1.5mm) although larger balloons can be required at very resistant proximal caps. Three case examples highlighting the successful use of BAM will be further described.

### Case 1

**Fig. (2):**
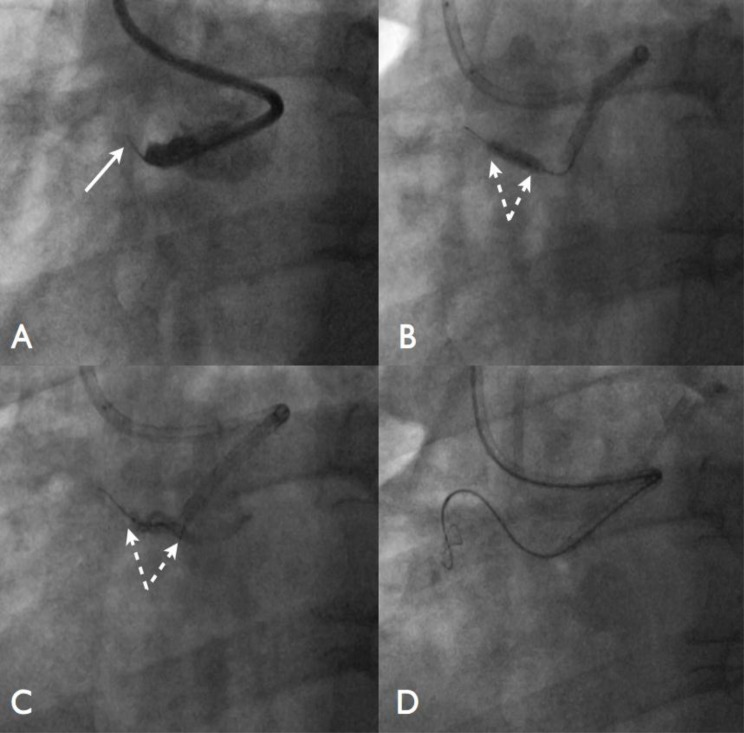
Panel A: There is a proximal occlusion of the right coronary artery. There are no branches to allow an anchor balloon to be sited. A ProVia 15g wire (Medtronic, Minneapolis, MN) has penetrated a short distance into the occlusion segment (arrow), although a Corsair will not pass beyond the cap. Panel B: A 2.5mm balloon is brought as close to the proximal cap and inflated. Panel C: The balloon is deliberately ruptured to created micro-dissection within the CTO segment. Panel D: This manoeuvre allows a knuckle wire and Corsair catheter to be advanced beyond the proximal cap into the CTO segment to facilitate a reverse CART.

A 57-year old hypertensive, diabetic male presented with a heavily calcified CTO of the proximal RCA having undergone previous PCI to the left circumflex (LCx). The chosen strategy was a primary retrograde approach with a 3D guide in the RCA and VL3.5 guide in the LCA. The heavily calcified proximal CTO cap was eventually penetrated with a ProVia 15g wire (Medtronic, Minneapolis, MN). It was not possible to advance any secondary equipment into the CTO. There were no proximal branches available to allow an anchor balloon to be sited. The patent proximal vessel was also too short to place an anchor balloon in this segment (Fig. **2**). In this case, a series of balloon were brought in as close to the proximal cap of the lesion as possible and ruptured at high pressure (1.2, 1.5, 2.0 and 2.5mm balloons). Eventually, micro-dissection was created within the CTO segment and it was possible to pass a Corsair and knuckle wire into the architecture of the vessel, eventually facilitating a successful reverse CART procedure.

### Case 2

**Fig. (3):**
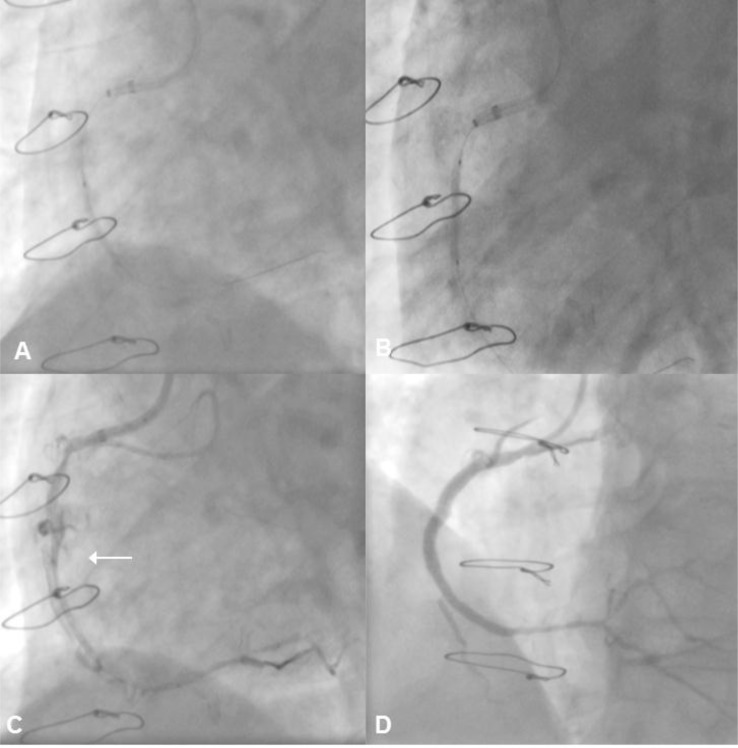
Panel A: There is a heavily calcified CTO of the proximal RCA, and use of a GuideLiner™ extension catheter is required to facilitate lesion crossing with a guidewire. Panel B: The lesion is successful crossed and a guidewire is advanced to the distal vessel. However, a range of balloons, a Finecross, and a Tornus micro-catheter (with anchor an anchor balloon), cannot be delivered to the distal vessel. Panel C: To allow delivery of secondary equipment to the vessel, intentional balloon rupture with controlled micro-dissection is performed (arrow). Panel D: This enables balloon and stent delivery to the distal vessel to aid successful revascularisation.

A 68-year old male with a previous CABG presented with a CTO of the RCA and an occluded saphenous vein graft (SVG) to this territory. There was no proximal cap ambiguity, the CTO length was < 20mm and the distal vessel was of good calibre. The chosen strategy was an antegrade approach. A Progress 200 wire was passed to the distal true lumen but secondary equipment could not be advanced beyond the distal portion of the occlusion segment despite the use of multiple semi-compliant balloons, a Finecross micro-catheter (Terumo, Japan), anchor balloons, a 2.6F Tornus (Asahi Intecc, Japan) micro-catheter and a Tornus plus anchor balloon. It was not possible to deliver any of the micro-catheters far enough into the lesion to facilitate passage of a rota floppy wire. Thus, BAM was performed at the point of obstruction using a 1.2 Minitrek balloon (Abbott Vascular, Santa Clara, CA) resulting in extensive intentional vessel dissection along a sub-intimal channel extending as far as the heart border. This facilitated successful balloon passage to the distal true lumen. Further pre-dilation allowed vessel reconstruction with sequential Nobori DES (Terumo, Japan) (Fig. **3**).

### Case 3

**Fig. (4):**
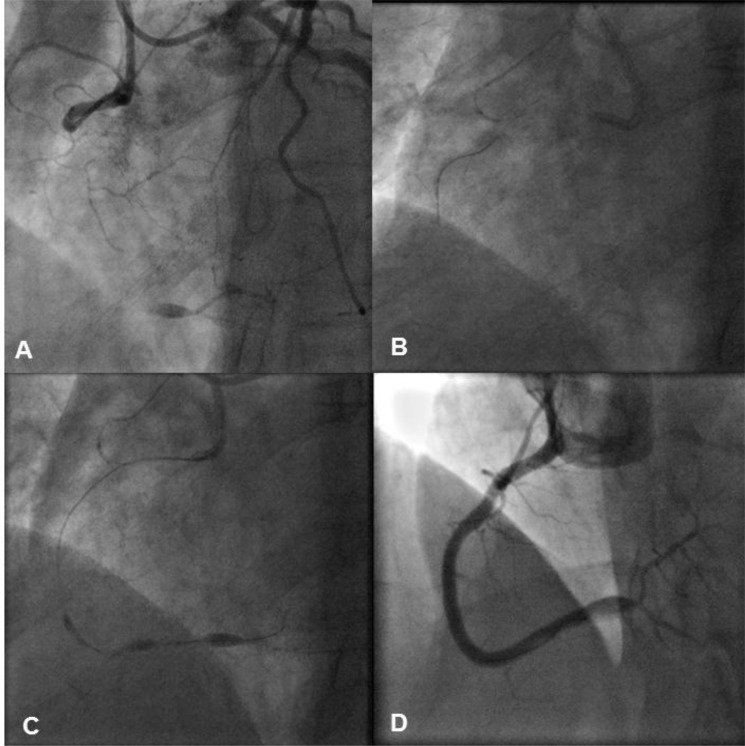
Panel A: There is a proximal CTO of the RCA, which is collateralised from the distal circumflex artery. Panel B: An antegrade Pilot 200 wire crosses the proximal lesion cap into the lesion. However, due to the resistant nature of the lesion, a range of semi-compliant balloons, a Corsair, and a CrossBoss™ catheter cannot be advanced any further. Panel C: Balloon assisted micro-dissection (BAM) is then performed by inflating a semi-compliant balloon at high-pressure in order to cause controlled dissection. This leads to immediate contrast passage into the distal true lumen. Panel D: As the distal true lumen has been re-entered with contrast, the vessel is re-wired, re-dilated and reconstructed with overlapping DES.

A 57-year old male with previous PCI to the LAD presented with refractory angina symptoms. There was a known CTO of the RCA with collateralisation from the distal circumflex artery. The chosen interventional strategy was an antegrade approach with an 8F AL 0.75 Guide. An antegrade Pilot 200 wire was used to enter the proximal lesion but semi-compliant balloons, a Corsair and a CrossBoss™ catheter (Boston Scientific, Natick, MA) would not cross the lesion. Thus, BAM was performed and this led to contrast passage directly into the distal true lumen. The distal vessel was then wired, pre-dilated and reconstructed with overlapping Promus Element stents that were post-dilated with a 4.0mm NC balloon (Fig. **4**). 

This demonstrates BAM plus contrast-guided re-entry as an option to facilitate successful recanalisation from within a CTO segment. A contrast-guided Sub-intimal Tracking and Re-entry (STAR) technique was developed as a modification of the original STAR method [19]. The STAR technique was first described by Colombo in 2005, and consists of creating a blunt sub-intimal dissection plane with a J-looped 0.014’ hydrophilic wire and a supporting OTW balloon, with subsequent wire manipulation to re-enter the vessel true lumen [20]. The clinical use of the technique was limited by less predictable results and the lack of angiographic guidance when creating a sub-intimal dissection plane. In the contrast-guided STAR technique, a CTO guidewire and an over-the-wire (OTW) balloon are used to penetrate the proximal cap of the lesion. The distal tip of the balloon is then inserted into the occlusive segment and the wire is withdrawn. Approximately 1–2mls of contrast are injected into the occlusion and confirmation of contrast within the vessel architecture or into the distal lumen facilitates further wire progression. Higher procedural success rates (82.3%) were seen with the contrast-guided STAR technique in a case series of 68 patients, with fewer perforations in comparison to the original STAR technique (4.4% vs. 9.7%) [19]. In this case, the BAM manoeuvre represents a higher pressure extension of the previously described contrast based STAR procedure. Whilst this is somewhat uncontrolled and may be unpredictable, BAM may lead to an acceptable outcome when no other options are available.

### Excimer laser coronary atherectomy (ELCA) and/or HSRA

ELCA (Spectranetics Corp, Colorado Springs, CO) is a useful adjunct in the management of severe fibro-calcific lesions following successful lesion crossing with a guidewire [21]. The ELCA catheters can be delivered with a standard 0.014” guidewire and are available in 0.9 mm, 1.4 mm, 1.7 mm, and 2.0 mm sizes. The 1.7 mm and 2.0 mm catheters are compatible with 7 F and 8 F guide catheters respectively while the 0.9 mm and 1.4mm can be used with 6 F guide catheters [22]. The 0.9 mm ELCA catheter is routinely used in CTO cases.

The ELCA delivers rapid ultraviolet B (UVB) light pulses to a coronary lesion. The light in the UVB region has a wavelength of 308 nm, with a shallow absorption depth of 50 microns. This shallow absorption depth limits medial and adventitial tissue damage in standard PCI. ELCA can emit high energy pulses lasting only a fraction of a second. The number of pulses emitted during a one second period is known as the ‘pulse repetition rate’. The duration of each pulse is termed a ‘pulse width’, which can be modified according to the nature of the treated lesion. Tissue breakdown via photo-ablation occurs in three steps. Firstly, rapid UV light absorption occurs resulting in severing of carbon-carbon bonds, with subsequent dissipation of energy. This energy dissipation leads to evaporation of intracellular water to produce a steam bubble that advances ahead of the laser catheter. Tissue breakdown occurs due to rapid expansion and contraction of these steam bubbles. The threshold energy required for the penetration of UV light into the surrounding tissue and the subsequent creation of a steam bubble is called ‘fluence’ (range: 30-80 mJ/mm^2^). High pulse energy delivery is more efficacious in managing calcified lesions. The resultant debris particles are<10 microns in diameter with minimal risk of distal embolization. 

Recently, data from a 4-year retrospective analysis of ELCA cases performed following balloon failure, has demonstrated ELCA to be an effective and safe adjunctive treatment. ELCA was used in 62% cases of balloon failure, with an overall procedural success rate of 91% [22]. There are cautionary notes to be made when considering ECLA as an adjunctive strategy. Its use should be limited only to cases of “true-lumen to true-lumen” antegrade wiring when the operator is certain the guidewire is intra-luminal. ELCA should not be used in cases where there is concern that the wire has taken a deep sub-intimal route. ELCA catheters are relatively indiscriminate in performing tissue ablation and will essentially ‘modify’ any tissue in their field of delivery (calcific / adventitia or otherwise). Thus, there is a greater risk of perforation compared to HSRA. The role of ELCA in the presence of very heavy lesion calcification is also more limited. If the lesion remains unexpandable, combined use of ELCA and HSRA can be performed in the hands of experienced operators familiar with the technology.

### c) ELCA

**Fig. (5):**
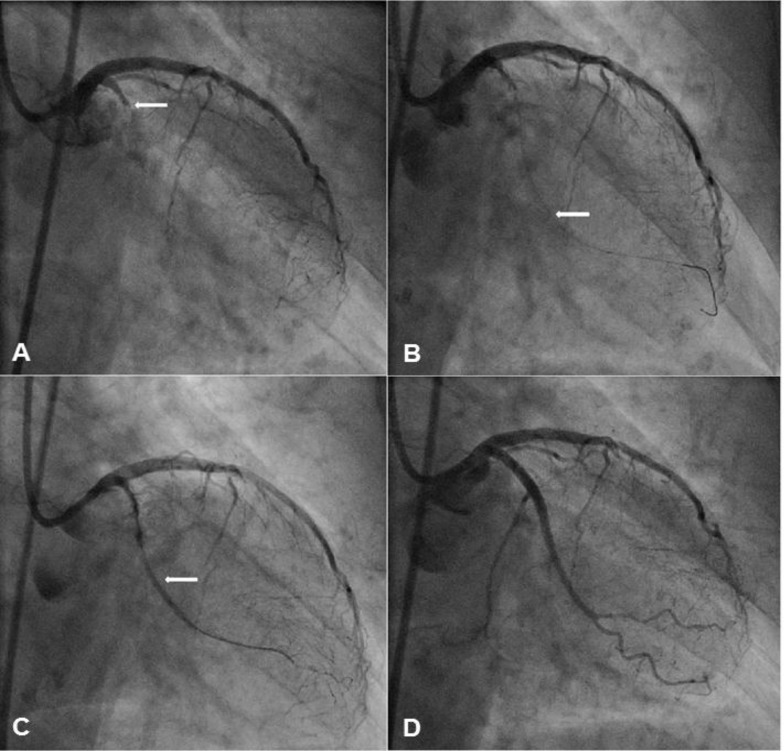
Panel A: There is a proximal CTO of the left circumflex artery with a visible stump and an unambigu ous proximal cap (arrow). Panel B: The proximal lesion cap is crossed with a Whisper MS guidewire that is advanced to the distal vessel. Panel C: The lesion could not be crossed despite use of multiple balloons, anchor balloons and a range of micro-catheters. Therefore, excimer laser coronary atherectomy (ELCA) is performed. Panel D: The vessel is reconstructed with overlapping DES.

Case example: A 77-year old woman with angina, preserved left ventricular (LV) systolic function presented with a known CTO of the left circumflex. Despite successful crossing of the lesion with a guidewire, micro-catheters and a range of balloons could not be advanced distally. Therefore, a 0.9 mm, X-80 ELCA catheter was used to deliver laser at a fluence of 60 mJ/mm^2^ (repetition rate 40 Hertz; 4400 pulses). After the laser catheter had crossed the lesion, the vessel was subsequently pre-dilated and reconstructed with overlapping DES (Fig. **5**).

### d) Combined ELCA and HSRA

This technique describes the use of ELCA followed by HSRA to facilitate delivery of secondary equipment where other adjunctive measures have failed.

**Fig. (6):**
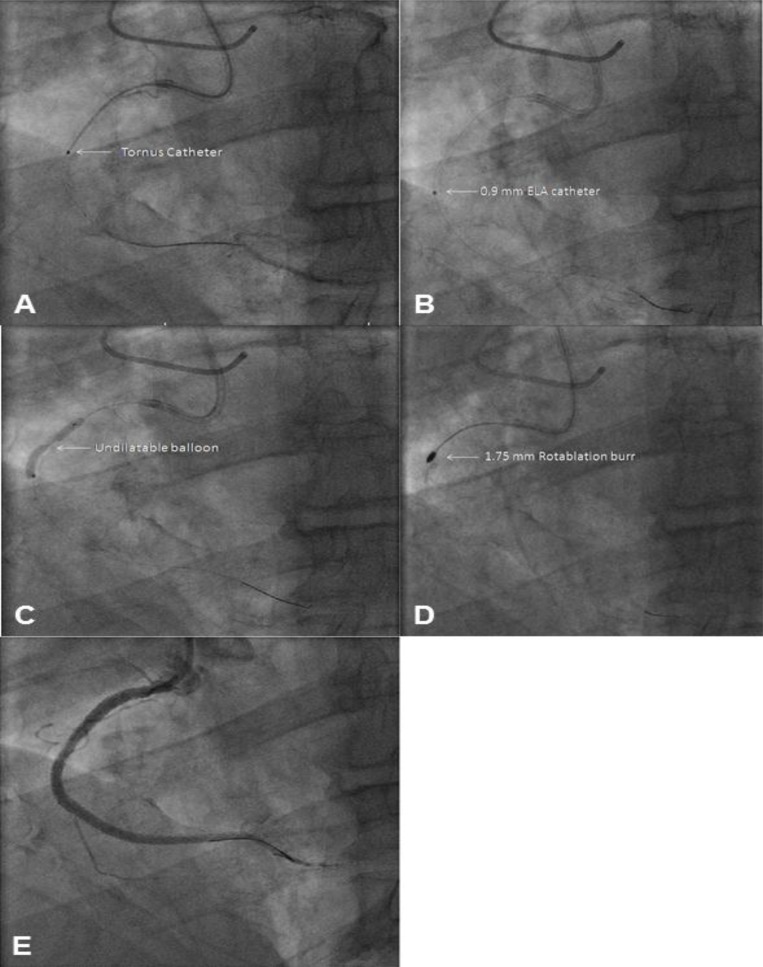
Panel A: There is a heavily calcified, resistant CTO in the proximal RCA. The lesion is crossed with a guidewire supported by a CrossBoss™ catheter, and the vessel is pre-dilated with a 1.2mm balloon. Progress of larger calibre balloons and a Tornus micro-catheter is unsuccessful due to the nature of the lesion (arrow). Panel B: At this stage, a 0.9mm X-80 ELCA catheter is used to modify this resistant lesion. Panel C: Following use of the ELCA, the vessel area is dilated again. However, the lesion remains resistant causing distortion of the balloon on inflation (arrow). Panel D: Due to the persistence of this lesion, a 1.75mm rotablation burr is used to modify the area. Panel E: The RCA is then reconstructed with overlapping DES back to the ostium.

Case example: A 68 year old male with refractory angina presented with a known RCA CTO. Cardiac MRI demonstrated ischaemia in the RCA territory. The chosen interventional strategy was an antegrade approach. A Pilot 200 wire was successfully advanced into the distal RCA beyond the CTO with assistance of a CrossBoss^TM^ CTO catheter. Despite pre-dilation with a 1.2mm balloon, further progress of larger calibre balloons and a Tornus were unsuccessful. Subsequently, a 0.9mm, X-80 ELCA catheter was used to deliver 5000 pulses at a fluence of 80mJ/mm^2^ (repetition rate: 80 Hz). This allowed passage of secondary equipment, although the lesion remained undilatable. Therefore, a 1.75mm HSRA burr was then used to facilitate balloon dilation of the proximal lesion. The entire vessel was the reconstructed with overlapping DES back to the ostium (Fig. **6**).

### e) HSRA to Modify a Stented But Resistant Lesion

**Fig. (7):**
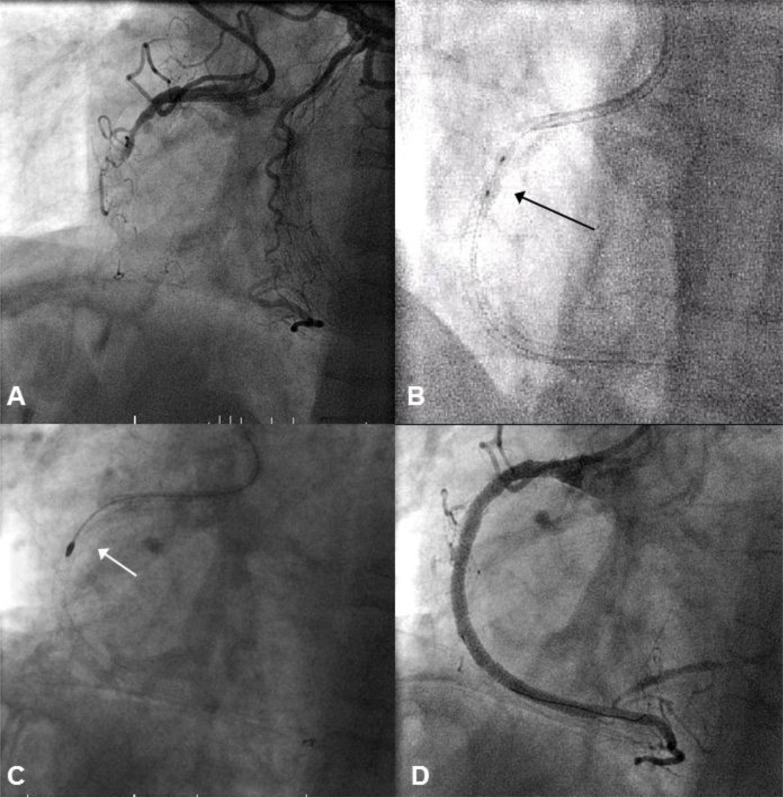
Panel A: There is a heavily calcified CTO of the RCA proximal to a previously deployed stent. A retrograde guidewire and Corsair could not be advanced due to extreme vessel tortuosity. Panel B: The lesion is crossed with an antegrade wire and pre-dilated. The vessel is reconstructed with overlapping Promus Element DES (2.5/38 and 3/38). A heavily calcified lesion persists at the proximal entry point to the previous RCA stent with obvious balloon distortion (arrow). Panel C: This undilatable area is modified with a 1.5 rotablation through the proximal portion of the newly deployed stent. Panel D: A further, short stent is deployed to overlap with the initial stents, and post-dilated with a 5.0 non-compliant balloon.

A 57 year old ex-smoker with a remote previous PCI to the proximal RCA represented with a proximal CTO of the RCA. There was some proximal cap ambiguity, the distal cap was unambiguous, the occlusion was >20 mm and the septal collaterals were useable. The chosen strategy was a primary retrograde approach. A retrograde wire was successfully progressed via the LAD to the distal RCA true lumen. However, a Corsair could not be advanced to the posterior descending artery (PDA) due to extreme septal collateral tortuosity. This necessitated a switch to an antegrade wire strategy. After wiring the distal vessel true lumen, pre-dilation was performed. The proximal RCA was heavily calcified proximal to the original stent. A 2.5/10 cutting balloon (Boston Scientific, Natick, MA) was unsuccessfully used to modify this area. The vessel was re-constructed using Promus Element stents and post-dilated with 3.0 and 3.5 mm non-compliant balloons. An undilatable lesion just proximal to the original stent became apparent. However, this lesion had already been covered with a new DES that was now under-expanded. A 1.5 burr was passed through this lesion into the proximal segment of the newly deployed stent. Further dilation with a 3.5 non-compliant balloon expanded this resistant lesion. This area was covered by a short segment of overlapping DES and the proximal vessel was post-dilated with a 5.0 non-compliant balloon back to the RCA ostium (Fig. **7**). The use of focused HSRA was unlikely to have caused significant stent disruption as the primary intention from the outset of the procedure was to cover the most proximal area with further stent deployment.

### f) HSRA Through a Heavily Calcified Sub-intimal Channel After Reverse CART

**Fig. (8):**
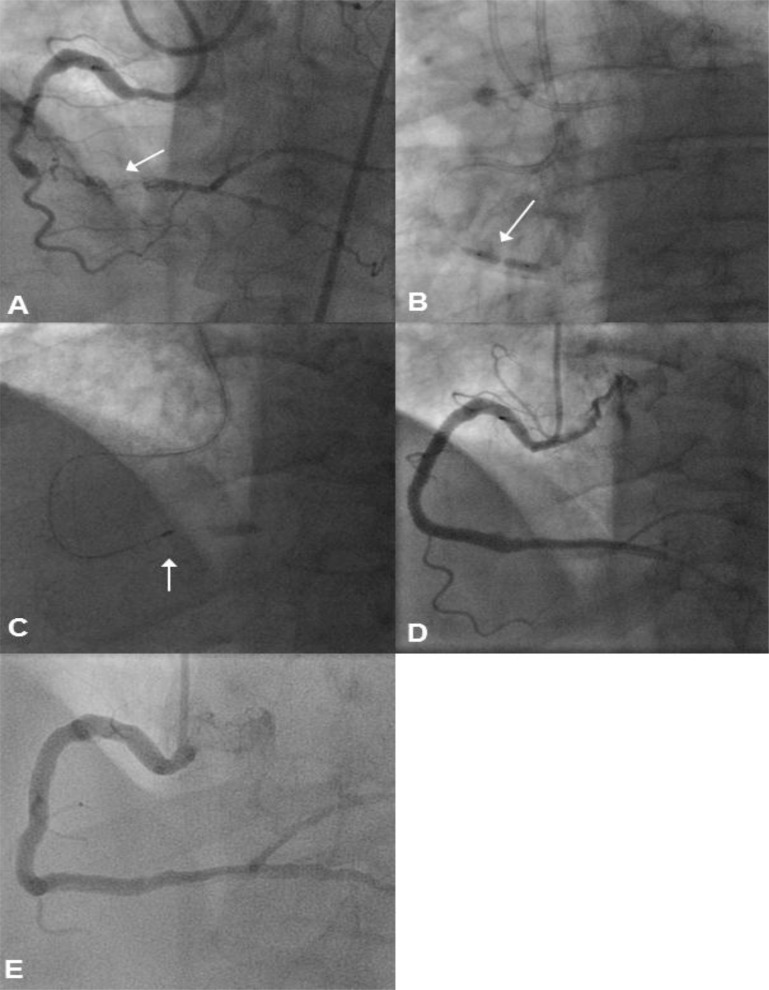
Panel A: There is a heavily calcified CTO of the RCA (arrow) that is supplied by collaterals from calcific LAD septal branches. Panel B: A reverse CART procedure has performed. After externalising a guidewire, antegrade balloon dilations with 2.5, 3.0, 3.5, and 4.0 balloons are performed but a resistant, focal lesion persists (arrow). Panel C: This resistant lesion is modified with a 1.25 burr (arrow). A large dissection plane is also evident in the distal segment of the RCA. Rotablation is safely performed through the surrounding dissection plane. Panel D: The vessel is reconstructed with overlapping DES. Panel E: Follow-up angiogram at 4-months demonstrating widely patent stents.

A 61 year old hypertensive, ex-smoker presented with exertional angina due to a calcified, tortuous CTO of the distal RCA. The proximal and distal caps were unambiguous, the occlusion was long and useable septal collaterals were present. Further to an unsuccessful antegrade wire approach, a retrograde approach was adopted. After delivering a Corsair to the distal vessel retrogradely, a Confianza Pro wire (Asahi Intecc, Japan) was used to penetrate the distal CTO cap. This was exchanged for a knuckled Pilot 200 wire which was advanced retrogradely to the mid RCA in the sub-intimal space. The “knuckle wire” technique is optimum for long segments of occlusion. The retrograde wire is manipulated to create a loop at the tip, which is advanced through the CTO segment (usually in the subintimal space). This manoeuvre can be performed safely without perforation of the vessel [3]. After completing a reverse CART, an RG3 guidewire was externalised. Further antegrade pre-dilation with 2.5, 3.0, 3.5 and 4.0 Apex balloons confirmed the persistence of a focal, highly resistant lesion. A large dissection plane was evident at the site of the reverse CART. This lesion required modification with a 1.25 burr that was safely passed through the sub-intimal space and the dissected distal RCA. The vessel was then reconstructed with DES and post-dilated to 4.0 mm. A follow-up angiogram after 4-months demonstrated widely patent stents (Fig. **8**).

### g) HSRA at a Dissected Graft Insertion Point

**Fig. (9):**
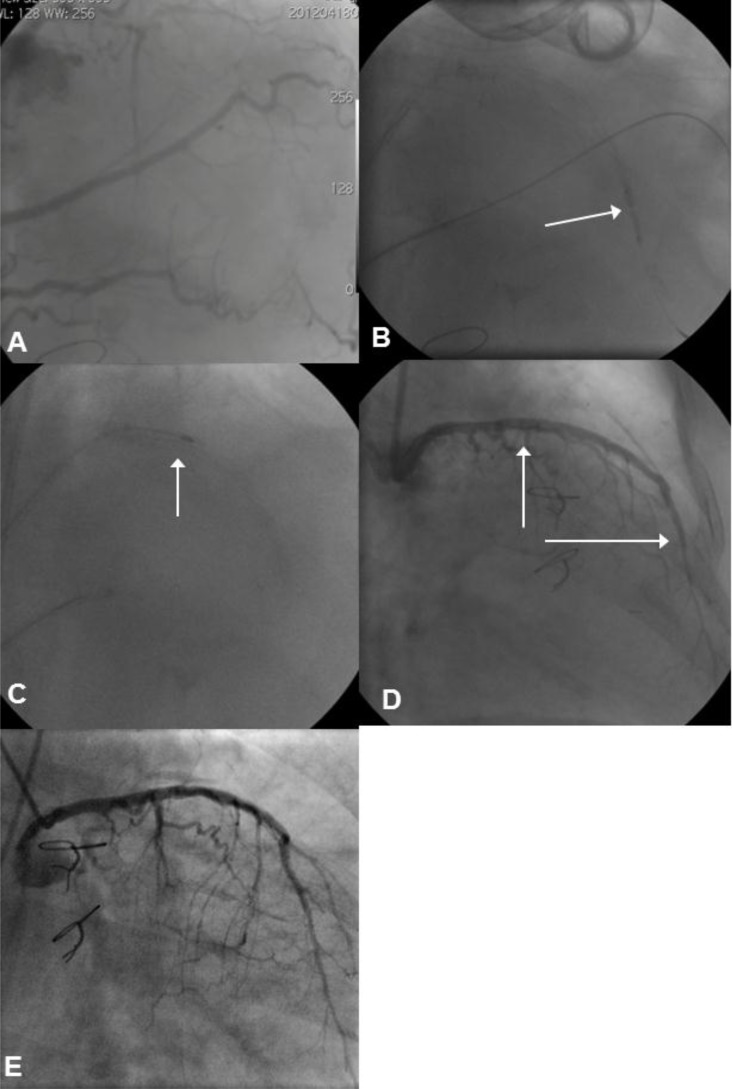
Panel A: There is a CTO of the native LAD with significant occlusive disease at the LIMA insertion point. Panel B: A retrograde Sion wire is advanced into the distal LAD lumen aided by a Corsair micro-catheter. A reverse CART procedure is performed after an antegrade knuckled Fielder XT wire was advanced to overlap with the retrograde wire. The LAD is then dilated and reconstructed with overlapping Promus Element DES back to the LMS. After stent deployment and high-pressure balloon inflation, a focal, resistant lesion is seen in the distal LAD at the LIMA insertion point (arrow). Panel C: This resistant lesion is modified with a 1.25mm burr (arrow), which is advanced through the long segment of stented LMS and LAD. Panel D: The final result following further stent deployment, post dilation and balloon angioplasty of the terminal vessel with a 1.5mm balloon. Panel E: Follow-up angiogram after 6-months.

A 79 year old female with previous CABG had intractable angina due to an occluded LAD and further occlusive disease at the insertion point of the left internal mammary artery (LIMA) graft which was non-functional. There was ambiguity of the proximal cap, the distal cap was unambiguous, the occlusion was long, and filled from epicardial collaterals off the PDA (via a patent SVG). A combined antegrade and retrograde approach was undertaken with the distal LAD true lumen accessed retrogradely via a Sion wire and a Corsair. After wire exchange, a knuckled Fielder XT (Asahi Intecc, Japan) was then passed through the CTO segment to the proximal cap of the occlusion. An antegrade knuckled Fielder XT was then overlapped with the retrograde wire within the CTO segment. A reverse CART was performed and an RG3 guidewire was externalised. The LAD was then pre-dilated with 1.5, 2.0 and 2.5 mm balloons. However, a focal area of resistant disease was apparent in the distal LAD at the LIMA insertion and this persisted despite high pressure balloon inflations and balloon rupture. The LAD was reconstructed with overlapping Promus Element DES from the distal LAD back to the left mainstem (LMS) ostium. The stented area was post-dilated to 2.5 mm, 3 mm, 3.5 mm and 4 mm, from the distal LAD to LMS. However, despite very high pressure post-dilation at the LIMA anastomosis, the calcific area persisted. This was modified with a 1.25 burr that was easily advanced through the long segment of stented LMS and LAD. The remaining vessel was reconstructed with a 2.25 mm DES and post-dilated to 2.5 mm. Follow-up repeat angiography at 6-months showed widely patent stents (Fig. **9**).

Historical practice has been to avoid using HSRA in the presence of intimal disruption and dissection. The current Rotablator^®^ Rotational Atherectomy System (Boston Scientific, Natick, MA) operating instructions cite use of the device in the presence of SVGs and angiographic evidence of dissection as contra-indicated. We have demonstrated successful use of HSRA through calcific diseased segments in the sub-intimal space. Rotablation preferentially modifies calcific segments with relative sparing of elastic sub-intimal and adventitial tissue. Use of complimentary ELCA in the setting of HSRA has also been demonstrated to be feasible and safe under appropriate circumstances.

## DISCUSSION

An increasing burden of resistant, calcific coronary lesions can be anticipated by the interventional cardiologist in future clinical practice. This reflects changing patient demographics with an elderly population and higher rates of chronic kidney disease, diabetes mellitus, and coronary graft failure. Thus, challenges with the successful revascularisation of such chronic occlusions can be anticipated. Standard techniques deployed to negotiate coronary lesions are unlikely to be successful alone. Thus, the operator should be prepared to acquire skills and gain understanding of a range of adjunctive techniques to facilitate successful CTO revascularisation. In the era of contemporary CTO PCI, high success rates can be anticipated in the hands of appropriately trained and experienced operators. Increased operator experience will enhance successful angiographic and clinical outcomes. The outcome of CTO revascularisation is dependent on clinical experience and, with a correlation between operator experience in general and CTO experience cases in particular [4]. This was reported in a study comparing technical success rates in 636 cases performed by retrograde operators (RO) and non–retrograde operators (NRO) of 58.95 vs. 75.2% for NRO vs. RO (p<0.0001). There were no differences in adverse outcomes between the two operator groups. Of note, the technical success rate of the RO group increased with time (up to 90%); whereas no change was seem in the NRO group, demonstrating that high CTO-specific operator case volume correlated with improved technical success rates. 

The challenges associated with CTO revascularisation can be broadly categorised into those related to lesion crossing, and those related to lesion modification. Novel technologies, wire strategies and device developments have complimented standard approaches to increase the success rates of CTO recanalisation. 

This article has reviewed and discussed the range of approaches that can be considered when faced with the challenge of resistant, calcific lesions. The cases demonstrate the successful implementation of these strategies in the recanalisation of heavily calcified CTOs. In addition, the authors have demonstrated the successful deployment of much more ‘extreme’ techniques to facilitate CTO revascularisation, where all other methods have failed. Such techniques should be considered only when other strategies have failed. In the cases described, several of the devices were used in a non-IFU setting, and thus, cannot be recommended as routine practice. 

In summary, in the contemporary era of CTO PCI, there is theoretically no exclusion to successful revascularisation. With an increasing burden of complex, calcific CTOs, a range of adjunctive strategies to facilitate successful recanalisation may need to be considered. Successful revascularisation correlates strongly with operator experience. The ranges of techniques described above are teachable and reproducible with increased operator experience and confidence.

## Figures and Tables

**Table 1.** Methods to facilitate lesion crossing.

Large active support catheters Anchor balloon techniques Extension catheters Terumo Heartrail II GuideLiner Micro-catheter technology (with / without anchor balloon support) Tornus Corsair Advanced wiring strategies Antegrade / retrograde wiring CART / Reverse CART

**Table 2.** Methods to facilitate lesion modification.

Very high-pressure balloon post-dilation Intentional balloon rupture / balloon assisted micro-dissection (BAM) Excimer coronary laser atherectomy (ECLA) Combined ECLA with high-speed rotational atherectomy (HSRA) High-speed rotational atherectomy (HSRA) alone
